# Temporal proteomic profiling reveals functional pathways in vaccinia virus-induced cell migration

**DOI:** 10.3389/fmicb.2023.1185960

**Published:** 2023-04-25

**Authors:** Jiayin Lu, Wei Liu, Xue-Zhu Chen, Yiwen Wang, Tianlei Ying, Liang Qiao, Yan-Jun Liu, Baohong Liu

**Affiliations:** ^1^Department of Chemistry, Shanghai Stomatological Hospital, Institutes of Biomedical Sciences, Shanghai Key Laboratory of Medical Epigenetics, International Co-laboratory of Medical Epigenetics and Metabolism (Ministry of Science and Technology), State Key Lab of Molecular Engineering of Polymers, Fudan University, Shanghai, China; ^2^MOE/NHC/CAMS Key Laboratory of Medical Molecular Virology, Shanghai Institute of Infectious Disease and Biosecurity, School of Basic Medical Sciences, Fudan University, Shanghai, China

**Keywords:** temporal proteomics, vaccinia virus, cell migration, host-virus interactions, regulation of actin cytoskeleton

## Abstract

**Introduction:**

Viral diseases have always been intricate and persistent issues throughout the world and there is a lack of holistic discoveries regarding the molecular dysregulations of virus-host interactions. The temporal proteomics strategy can identify various differentially expressed proteins and offer collaborated interaction networks under pathological conditions.

**Method:**

Herein, temporal proteomics at various hours post infection of Vero cells were launched to uncover molecular alternations during vaccinia virus (VACV)-induced cell migration. Different stages of infection were included to differentiate gene ontologies and critical pathways at specific time points of infection via bioinformatics.

**Results:**

Bioinformatic results showed functional and distinct ontologies and pathways at different stages of virus infection. The enrichment of interaction networks and pathways verified the significances of the regulation of actin cytoskeleton and lamellipodia during VACV-induced fast cell motility.

**Discussion:**

The current results offer a systematic proteomic profiling of molecular dysregulations at different stages of VACV infection and potential biomedical targets for treating viral diseases.

## 1. Introduction

The sudden large-scale monkeypox virus outbreak has put enormous stresses on public health infrastructures, and more than 12,000 people have been infected since early May 2022 (Osterholm and Gellin, 2022). Studies of the pathogenesis of the virus infection are necessary and urgent for further drug developments. Vaccinia virus (VACV) is a large double-stranded Orthopoxvirus prototype, which shares symptoms and virus dissemination similar to monkeypox (Realegeno et al., 2017). VACV infection can cause variable cytopathic effects (CPEs) including actin tail formation, plaque formation, and virus-induced cell motility (Cudmore et al., 1995; Strauss, 1996) that facilitate viral dissemination.

Cell migration is a morphological and biological process driven by different environmental cues (Le Berre et al., 2013; Liu et al., 2015; Bangasser et al., 2017; Kameritsch and Renkawitz, 2020) or chemotaxis (Tiberio et al., 2018; Karnezis et al., 2019; Um et al., 2019). Virus infection can induce profound changes in cell metabolism (Fritsch and Weichhart, 2016; Girdhar et al., 2021) and motility (Valderrama et al., 2006; Baasch et al., 2021) to enlarge the scale of viral diffusion. Single viral particles extend and approach adjacent cells on the tip of actin tails (Cudmore et al., 1995). Early vaccinia viral genes have been reported as necessary and sufficient factors to induce cell migration (Sanderson et al., 1998). The small guanosine triphosphatase kinase (GTPase) Rho family-mediated regulation of cell contraction and actin-dependent cell motility was the most recognized mechanism of VACV-induced cell migration (Arakawa et al., 2007a,b; Durkin et al., 2017). Researches on virus-host interactions have indicated that the viral protein F11-mediated RhoA signaling inhibited virus release concomitant and reduced virus-induced cell migration (Handa et al., 2013). However, a comprehensive proteomic profiling of intracellular proteins without presupposed hypothesis has always been necessary for understanding holistic cellular changes during virus-induced fast cell migration.

Proteomic analysis is considered as a promising strategy to get a global view of complex biological processes, taking advantage of high-throughput profiling (Cui et al., 2022) and extensive dynamic ranges (Canterbury et al., 2008). Recently, liquid chromatography-mass spectrometry (LC–MS)-based proteomics have been utilized for investigating biomarkers in various biological samples, such as extracellular vesicles (Wu et al., 2023), biological tissues (Li et al., 2022), serum (Zhang et al., 2022), and paraffin-embedded tissues (Cai et al., 2022), indicating wide clinical applications regarding cancers and diseases. Rapidly developed proteomic technology provides a more precise and more comprehensive insight into virus or bacterial infections (Grossegesse et al., 2020). Soday et al. identified histone deacetylase 5 (HDAC5) as a host antiviral factor by generating highly temporal multiplexed proteomics (Soday et al., 2019). Kok et al. concluded that single-cell omics strategies, including proteomics, could offer unprecedented opptunities for unraveling infection stages of single virus and heterogenous host cells (Kok et al., 2017). Also, Schrad et al. identified specific proteins released from capsids during the initial stages of giant virus infection through proteomics (Schrad et al., 2020). Generally, the rigorous and extensive proteomic profiling provides a high-throughput approach to explore protein–protein interaction networks and functional pathways simultaneously. Moreover, temporal proteomics at multiple time points of complicated biological processes can offer intuitive trends of intrinsic alternatives and molecular ontologies.

**SCHEME 1:**
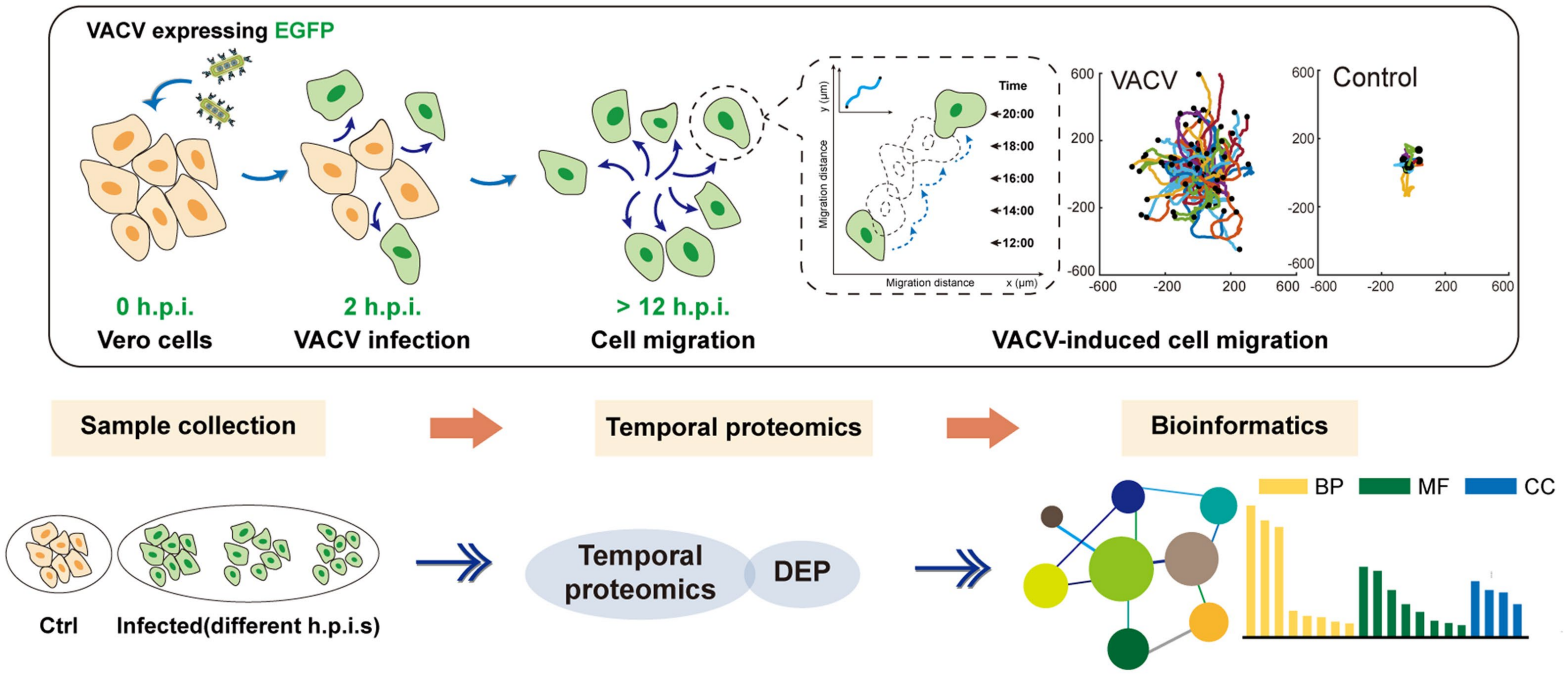
The holistic workflow of temporal proteomic profiling of VACV-infected Vero cells. h.p.i., hours post infection; VACV, vaccinia virus; EGFP, enhanced green fluorescent protein; DEP, differentially expressed proteins; BP, biological process; MF, molecular function; and CC, cellular components.

Herein, we launched a temporal quantitative proteomics approach combined with real-time live-cell imaging to discover the molecular changes in virus-host interactions (Scheme 1) at different hours post infection (h.p.i.). Based on protein identification and subsequent bioinformatic analysis, we focused on regulation of actin cytoskeleton, biosynthesis of amino acids, fluid shear stress and atherosclerosis. Gene Ontology (GO) enrichment highlighted various terms at different stages of virus infection. The successfully acquired data profiled temporal proteomic alternations during VACV-induced cell migration, thus supplementing previously reported mechanisms of virus-host interactions. The systematic proteomic analysis also provided potential therapeutic biomarkers and biomedical applications for viral treatments.

## 2. Materials and methods

### 2.1. Chemicals and reagents

Deionized (DI) water (18.2 MΩ·cm) was produced by a Milli-Q system (Millipore, Bedford, MA, United States) and used in all experiments. HPLC grade acetonitrile (ACN) and methanol as well as analytical grade acetone and hydrochloric acid (HCl, 37%) were purchased from Sinopharm Chemical Reagent Co., Ltd. (Shanghai, China). Analytical grade formic acid was purchased from J&K Scientific Ltd. (Beijing, China). Iodoacetamide (IAA), trizma base, urea, sodium dodecyl sulfate (SDS), and ammonium bicarbonate (NH_4_HCO_3_) were purchased from Sigma-Aldrich (St. Louis, MI, United States). Bond-breaker TCEP solution (0.5 mol/L) and protease inhibitor cocktail (100×, EDTA-free) were purchased from Thermo Fisher Scientific (Rockford, United States). Trypsin used for protein digestion was bought from Hualishi Technology Co., Ltd. (Beijing, China). Trypsin–EDTA solution (0.25%, with phenol red) used for cell digestion and phosphate buffered saline (PBS, 1×) were purchased from Solarbio Science & Technology Co., Ltd. (Beijing, China).

### 2.2. Cell culture and vaccinia virus

Vero cells were cultured in DMEM (GIBCO) supplemented with 10% fetal bovine serum (FBS, GIBCO), and 100 U/mL penicillin and streptomycin (GIBCO) at 37°C and 5% CO_2_. For VACV multiplication, the monolayer of Vero cells was infected with VACV for 72 h in DMEM containing 2% FBS. After freeze-thawing three times, the lysates were centrifuged at 15,000 rpm at 4°C for 10 min to get supernatants. The multiplied VACV titer was quantified according to the TCID_50_ (Median Tissue Culture Infectious Dose) assay.

To seed experimental cell candidates, trypsin–EDTA solution was added to suspend adherent Vero cells. Then 150 μL of cell suspension containing 1 × 10^5^ Vero cells was seeded into each well of a six-well plate and the extra 1.8 mL of cell culture medium was added immediately with gentle shakes. After the overnight incubation, the cell culture medium was removed and equal volumes of VACV-expressing enhanced green fluorescent proteins (EGFP) were added to infect monolayer cells at a multiplicity of infection (MOI) of three. Biological triplicates were cultured in the same batch.

### 2.3. Live cell imaging

Time-lapse images were acquired with 40× objectives using a DMi8 inverted microscope (Leica) equipped with an ORCA-Flash4.0 camera (Hamamatsu Photonics) controlled by MetaMorph software (Universal Imaging) in a cage incubator (Okolab).

### 2.4. Temporal proteomics

Vero cells infected with VACV at 12, 24, and 36 h.p.i. and cells without treatment were harvested by 500 μL trypsin–EDTA free digestion. Subsequently, cells were washed with 1 × PBS and centrifuged three times at 1,000 rpm, 4°C, to gain cell pellets (about 6 × 10^5^ cells per plate). Then cell pellets were rapidly transferred into a 1.2 mL Eppendorf tube for later sample preparation. For proteomic analysis, 400 μL of lysis buffer (20 mmol/L Tris, 8 mol/L Urea, 1% SDS, HCl, and pH = 8–9) and 4 μL of 100× protease inhibitor cocktail were added for cell lysis. After a thorough cell disruption by ultrasonication for 10 min, the supernatant was obtained by centrifuging at 15,000 rpm, 4°C, and stored at −80°C. Pierce BCA protein assay (Thermo Fisher Scientific, Rockford, United States) was utilized to quantify protein concentrations. After protein quantification, 150 μg of protein was carbamidomethylated by 10 mmol/L TCEP (final concentration) for 1 h under 37°C and 40 mmol/L IAA (final concentration) for 45 min in the dark at 25°C in 25 mmol/L ammonium bicarbonate (ABC) solution with the protein concentration of 1–2 μg/μL. Six times volumes of acetone (pre-cooled under −20°C) were added to precipitate protein pellets. Subsequently, proteins pellets were centrifuged, washed with 90% acetone three times to remove impurities, digested by adding 4 μL of trypsin with a trypsin:protein ratio of 1:50 (w/w), and incubated at a mixing speed of 600 rpm/h overnight at 37°C.

After the overnight digestion, the supernatant was desalted with desalting columns (MonoSpin C18, GL Science, Inc., Tokyo, Japan) using standard protocols in the kit. The obtained supernatant was then dried in a vacuum drying system (LNG T98 vacuum dryer, Taicang Huamei Biochemical Instrument Factory, Jiangsu, China) at room temperature for 1 h. Dried peptides were resuspended in DI water and quantified using Pierce Quantitative Colorimetric Peptide Assay (Thermo Fisher Scientific, Rockford, United States). Quantified peptides were lyophilized at −40°C overnight and stored at −80°C.

### 2.5. Nano-UPLC-MS/MS analysis

The iRT kit (Ki3002, Biognosys AG, Switzerland) was added to all the samples to calibrate the retention time. Then, all samples were analyzed by online nano flow liquid chromatography tandem mass spectrometry performed on an EASY-nanoLC 1200 system (Thermo Fisher Scientific, MA, United States) connected to a Orbitrap Q-Exactive HF-X mass spectrometer (Thermo Fisher Scientific, MA, United States) by Shanghai Omicsolution Co., Ltd. Three μg peptides of each sample were loaded and analyzed in data independent acquisition (DIA) mode. The survey of full scan MS spectra (m/z 350–1,500) was acquired in the Orbitrap with 60,000 resolution. All precursor ions were entered into collision cell for fragmentation by higher-energy collision dissociation (HCD), and the collision energy was 28. The MS/MS resolution was set at 30,000.

### 2.6. Statistical analysis and bioinformatics of temporal proteomics

Raw data of DIA were processed and analyzed by Spectronaut 15 (Biognosys AG, Switzerland) with default settings and retention time prediction type was set to dynamic iRT. The database was downloaded from UniProt (https://www.uniprot.org/taxonomy/60711, Accessed on June 28, 2021, containing 19,229 proteins). Q-value (FDR) cutoff on precursor level and protein level were both 1%. All selected precursors passing the filters were used for quantification. MS2 interference removed all interfering fragment ions except for the three least interfering ones. The average top three filtered peptides which passed the 1% Q-value cutoff were used to calculate the major group quantities.

The identified protein groups were filtered and duplicates were removed before further statistical analysis. Each protein group quantity was calculated by the average of protein raw intensity. Fold changes (FC) calculated by protein raw intensities and *t*-tests for *p* values were implemented. FC > 2 and *p* value <0.05 were considered as thresholds to identify differentially expressed proteins (DEPs). The GO enrichment and pathway enrichment were done using the database for annotation, visualization, and integrated discovery (DAVID, https://david.ncifcrf.gov/) and the Kyoto encyclopedia of genes and genomes (KEGG, https://www.kegg.jp/), respectively. The protein–protein interaction network was analyzed with the search tool for recurring instances of neighboring genes (STRING, https://cn.string-db.org/). The heatmap and principal component analysis (PCA) were launched by complexheatmap and factoextra R package (http://bioconductor.org/, accessed on January 20, 2022), respectively.

### 2.7. Analysis of cell trajectories

For cell motility analysis, the nucleus images labeled by Hoechst were tracked using the TrackMate plugin in FIJI. Briefly, the calibrated-image sequences were detected by a log detector and were tracked by a simple LAP tracker. The spots in tracks statistics were further analyzed by self-written code in Matlab to obtain the normalized cell trajectories.

## 3. Results and discussion

### 3.1. VACV infection induces faster Vero cell migration

**Figure 1:**
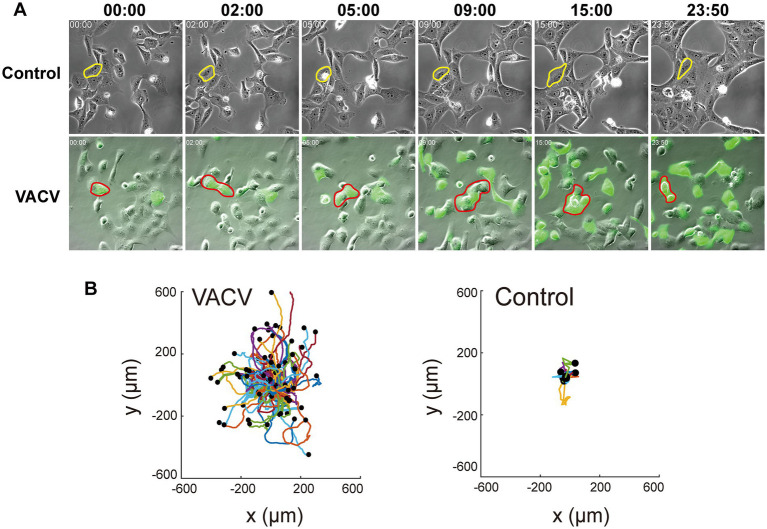
VACV infection induces faster Vero cell migration. **(A)** The images at different time points of the non-infected and VACV-infected Vero cells within 24 h.p.i. One of the typical cells in each group has been circled to clarify the movement. **(B)** The cell trajectory analysis of non-infected and VACV-infected Vero cells at 24 h.p.i.

VACV-infected Vero cells can initiate fast cell migration to spread progeny virions, thus causing viral dissemination efficiently. To comprehensively understand intrinsic molecular functions during this process, we performed VACV infection assay of Vero cells at a MOI of three with three biological replicates. We chose three different time points, 12, 24, and 36 h.p.i., to discover the early and late stages of proteomic variables. A real-time live-cell imaging system of non-infected and infected Vero cells expressing EGFP was implemented to confirm phenotypic differences. We observed VACV-induced fast cell migration compared to non-infected host cells unambiguously (Figure 1A). The specific typical cell was outlined for cell motility characterization in each group. VACV-infected host cells undoubtedly performed faster cell migration with changing morphologies. The analysis of cell trajectories summarized that cell movements based on nuclear locations showed significant disparity between non-infected and VACV-infected cells (Figure 1B). Normal epithelial cell migrated within 400 μm of tracking ranges and VACV-induced infected cells moved in ranges that were three times as extensive.

**Figure 2:**
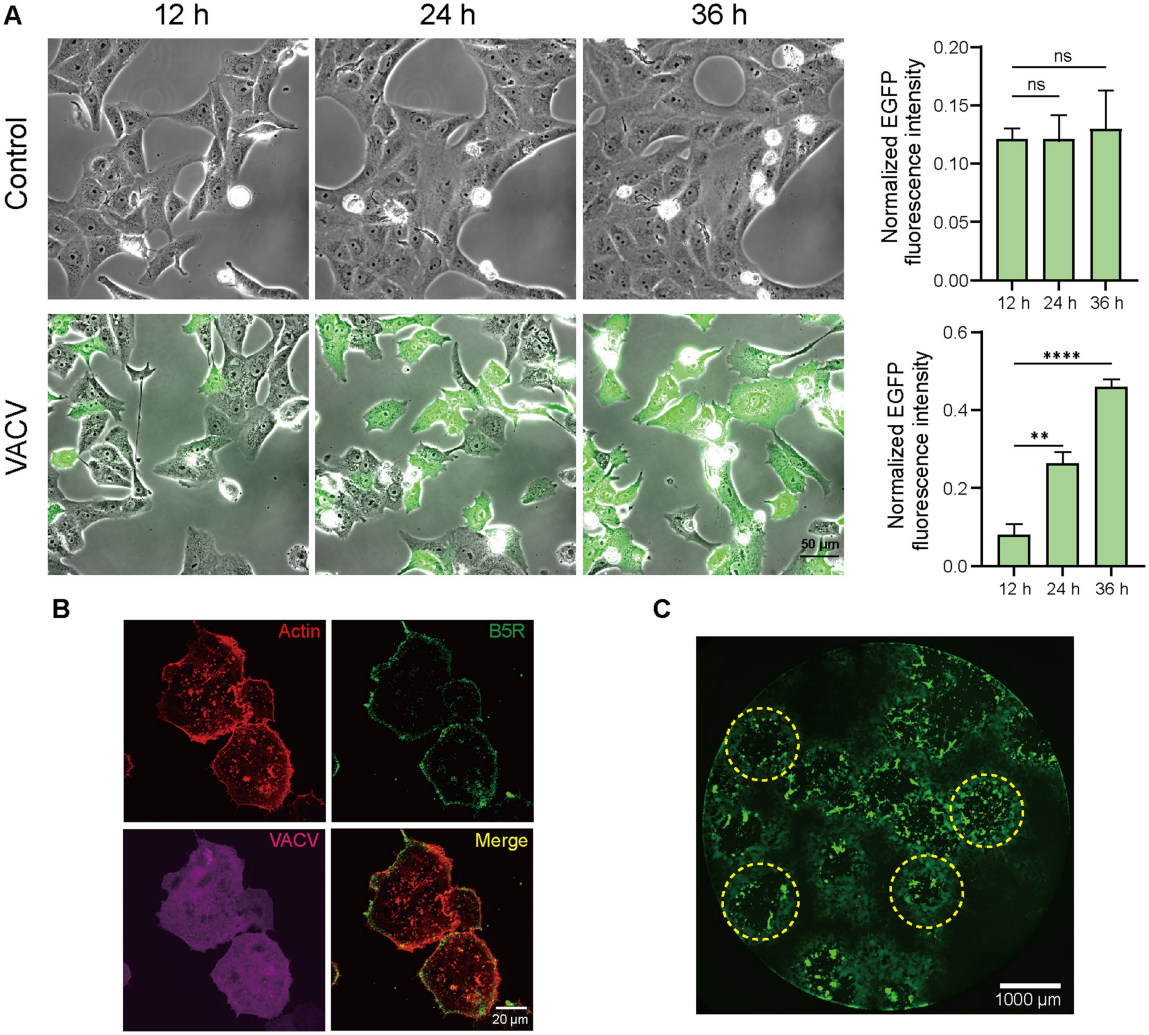
High-resolution live-cell images of VACV-infected host cells. **(A)** Representative images and normalized fluorescent quantitative analysis of non-infected and VACV-infected Vero cells (EGFP label) at 12, 24, and 36 h.p.i. Scale bar represents 50 μm. Data are mean ± SEM. ^*^p < 0.05, ^**^p < 0.01, and ^***^p < 0.001 (unpaired *t*-test); ns, not statistically significant. **(B)** Representative fluorescence images of VACV-infected cells containing actin tails (Actin: red; B5R: green; VACV: magenta). Scale bar represents 20 μm. **(C)** Representative fluorescence images of virus plaques (yellow dotted circles) at VACV 48 h.p.i. Scale bar represents 1,000 μm.

The infection level of host cells increased significantly due to viral dissemination and virus-induced cell motility. Fluorescent images of infected Vero cells expressing EGFP directly informed infection level accumulation. Quantitative normalized EGFP fluorescence intensity indicated that the infective level at 36 h.p.i. was over four times of that at 12 h.p.i. (Figure 2A). Except for VACV-induced fast cell motility, other CPEs like actin tail formation (Figure 2B) and plaque assay (Figure 2C) also occurred during virus infection. To gain a better overall understanding of molecular mechanisms during VACV-infected cell migration, we further launched systematic temporal proteomic profiling.

### 3.2. Qualitative and quantitative proteomic analysis of VACV-induced cell migration

**Figure 3:**
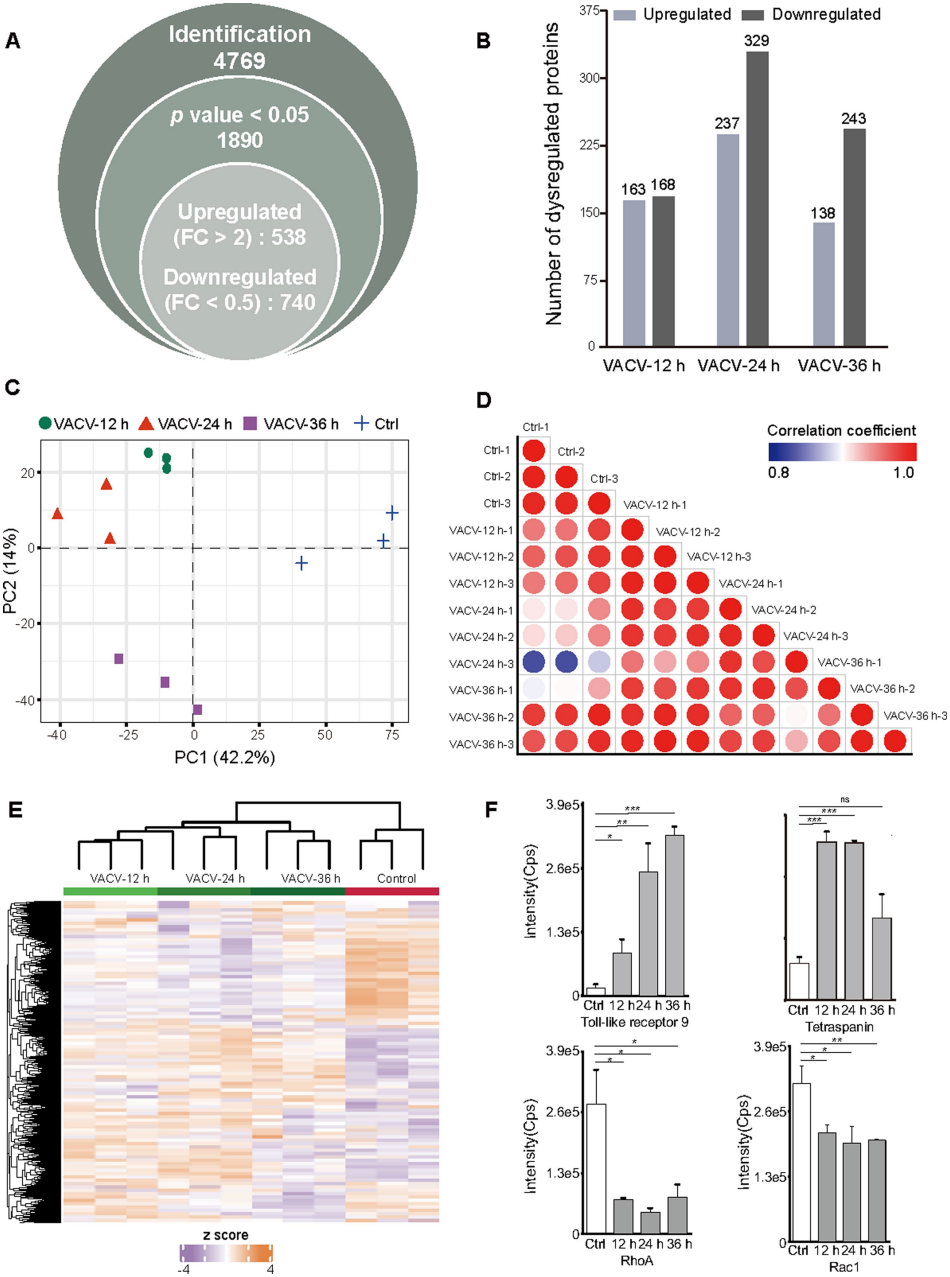
Temporal proteomic qualitative and quantitative analysis of VACV-induced cell migration. **(A)** The number of identified protein groups, significantly expressed proteins under the threshold of *p* value <0.05, and upregulated and downregulated proteins under the threshold of fold change >2. **(B)** The number of quantitatively upregulated and downregulated proteins at different time points of infection (12, 24, and 36 h.p.i.) under the thresholds of value of *p* < 0.05 and fold change > 2, respectively. **(C)** The principal component analysis (PCA) plot based on temporal proteomics of non-infected cells and VACV-infected cells at different time points of infection. **(D)** The sample correlation plot of non-infected and VACV-infected Vero cells. The color shade represents coefficients between these groups. Each group has three biological replicates. **(E)** The heatmap of dysregulated proteins under the criteria of *p*  value < 0.05 and fold change > 2 of non-infected and VACV-infected Vero cells. Each group has three biological replicates. **(F)** The intensities of immune-associated proteins, including toll-like receptor 9 and tetraspanin, and actin cytoskeleton regulation-associated proteins, including RhoA and Rac1. Data are mean ± SEM. ^*^p < 0.05, ^**^p < 0.01, and ^***^p < 0.001 (unpaired *t*-test); ns, not statistically significant.

The non-infected and VACV-infected Vero cells at a MOI of three at 12, 24, and 36 h.p.i. were cultured and harvested for temporal proteomics, respectively. Protein identification and quantification were performed using a direct-DIA strategy. The systematic quantitative proteomic profiling extensively screened the temporal proteome changes during VACV infection. In total, 4,769 protein groups were identified totally at three different time points of infection after redundancy filtering and removal of duplicates (Supplementary Dataset S1). Over 25% of identified proteins were quantitatively considered as significantly dysregulated proteins under the criteria of *p* value <0.05 and FC > 2, including 538 upregulated and 740 downregulated proteins (Figure 3A). Comparing the number of dysregulated proteins across various time points, it was found that the dysregulation in the proteome reached maximum at 24 h.p.i. (Figure 3B). The PCA plot thoroughly separated the control group and infection groups over different time points of infection (Figure 3C). The proteome sample correlation coefficients were also calculated (Figure 3D), which clearly illustrated that the proteome at 24 h.p.i. was the most significantly different from the non-infected group. Hence, 24 h.p.i. was emphasized as the most extensive molecular dysregulation time point among the three time points of infection.

Dysregulated protein expressions were shown by the heatmap (Figure 3E). Among these proteins, the small GTPase Rho family associated with actin cytoskeleton regulation (Warner et al., 2019) and proteins associated with the immune responses of host cells, such as toll-like receptors and tetraspanins, were quantitatively identified (Figure 3F). RhoA and Rac1, which are crucial mediators of cell polarity, were significantly downregulated. Toll-like receptors responsible for eliciting innate immune responses and cell motility (Xun et al., 2021) were upregulated after infection, and tetraspanins as functional regulators for invasive virus or bacterial infections (Karam et al., 2020) were also upregulated, indicating the activation of immune responses of host cells after virus entry. Matching intuitive morphologies of infected cells with intrinsic molecular alternations can better understand intracellular mechanisms during VACV-induced cell migration.

### 3.3. VACV hijacked functional pathways over various infection stages to accelerate cell motility

**Figure 4:**
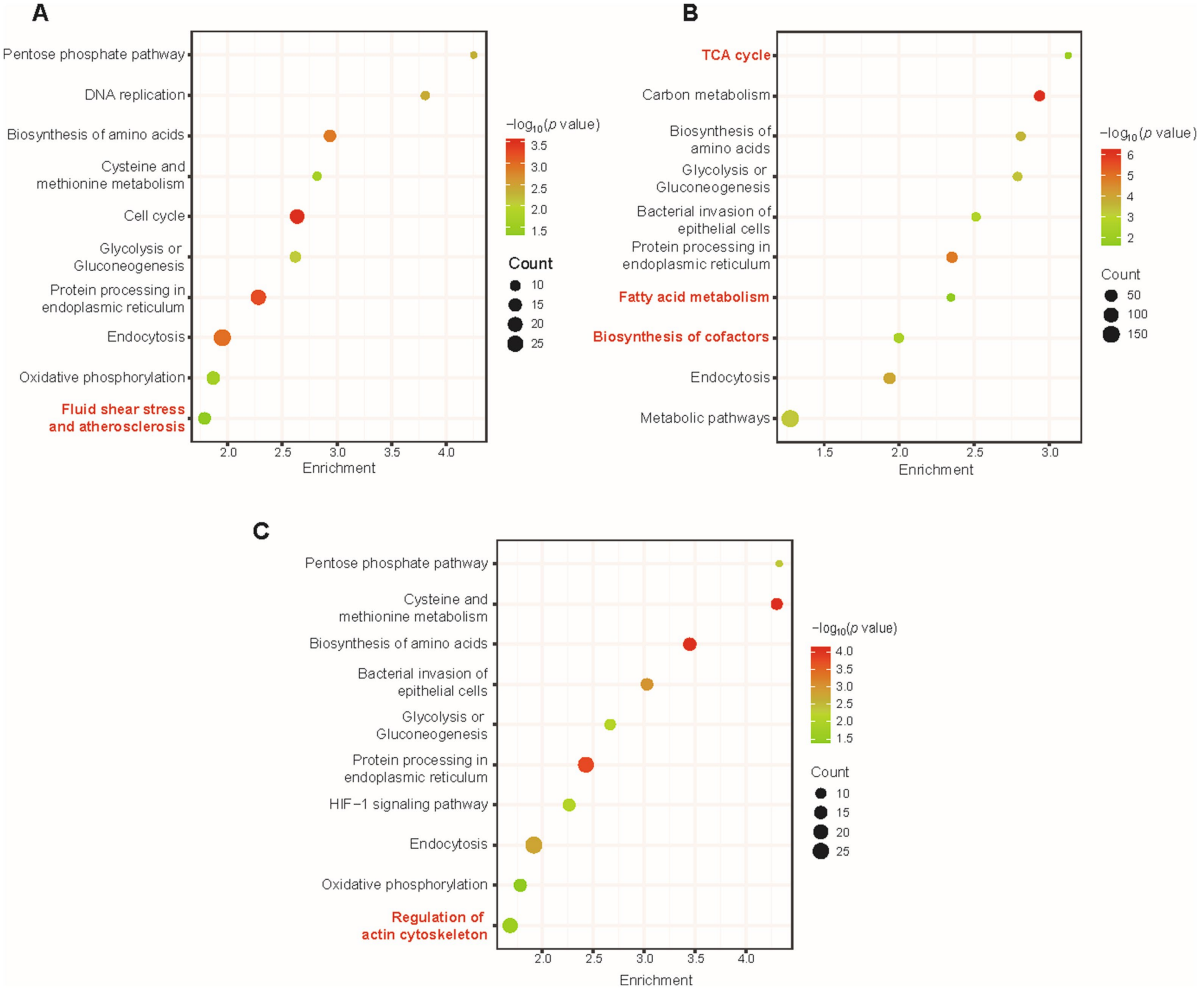
The overall KEGG pathway enrichment during VACV-induced cell migration at multiple time points of infection. Pathway enrichment of dysregulated proteins at 12 h.p.i. **(A)**, 24 h.p.i. **(B)**, and 36 h.p.i. **(C)**. Gradient color represents *p* value, and the size of circles represents protein counts in each pathway. Significant terms are marked in red.

Bioinformatic statistical analysis was further launched to analyze the proteome data in order to enrich the metabolic pathways from the proteome data and to find the differences in enriched pathways among different virus infection conditions. The enrichment of KEGG pathways and GO launched by the DAVID database verified distinctive biological terms between different stages of virus infection. By calculating *p* values, significant KEGG pathways at different h.p.i.s under the criteria of *p* value <0.05 were enriched and shown in colored bubble diagrams. Commonalities among the three time points of infection were biosynthesis of amino acids, glycolysis or gluconeogenesis, endocytosis, and protein processing in endoplasmic reticulum (Figures 4A–C). Metabolic reprogramming was manipulated by invasive pathogens and consistent with physiopathology. For instance, VACV has been reported to fulfill efficient viral protein synthesis with high concentration of asparagine (Pant et al., 2019). Also, Thai et al. found that adenovirus promoted host cell glucose metabolism compared to non-infected cells (Thai et al., 2014). Additionally, Fischer et al. discovered a pivotal role of endocytosis in the biological activity of the F protein cytoplasmic domain of Cedar virus, which is responsible for the signal transduction from cell surfaces (Fischer et al., 2020). Similarly, VACV hijacked host metabolic cycles for the high demands of abnormal cellular biological manners.

At various time points of infection, there was distinguished enrichment of different KEGG terms, in agreement with specified stages of VACV infection. At 12 h.p.i., dysregulated proteins led to fluid shear stress and atherosclerosis (Figure 4A), which has often been emphasized in mechanosensitive signaling research of diverse systems biology, especially in complex extracellular environments (Souilhol et al., 2020). At 24 h.p.i., tricarboxylic acid cycle (TCA cycle), fatty acid metabolism, and biosynthesis of cofactors were significantly enriched (Figure 4B). Regular metabolic alternations were activated and dysregulated for available energetic demands of virus production (Zandi et al., 2022). At 36 h.p.i., regulation of actin cytoskeleton was enriched and distinguished from other time points of infection (Figure 4C). At the early stage of virus infection, DNA replication and biosynthesis of small molecules were necessary for the viral life cycle and protein synthesis. To further enlarge infection spread and promote viral dissemination, VACV initiated faster host cell migration by regulating actin cytoskeleton and small GTPase Rho family expressions. Temporal proteomics provided consequent metabolic pathway changing tendencies with unbiased large-scale proteomic profiling and highlighted multiple factors responsible for accelerating cell motility.

**Figure 5:**
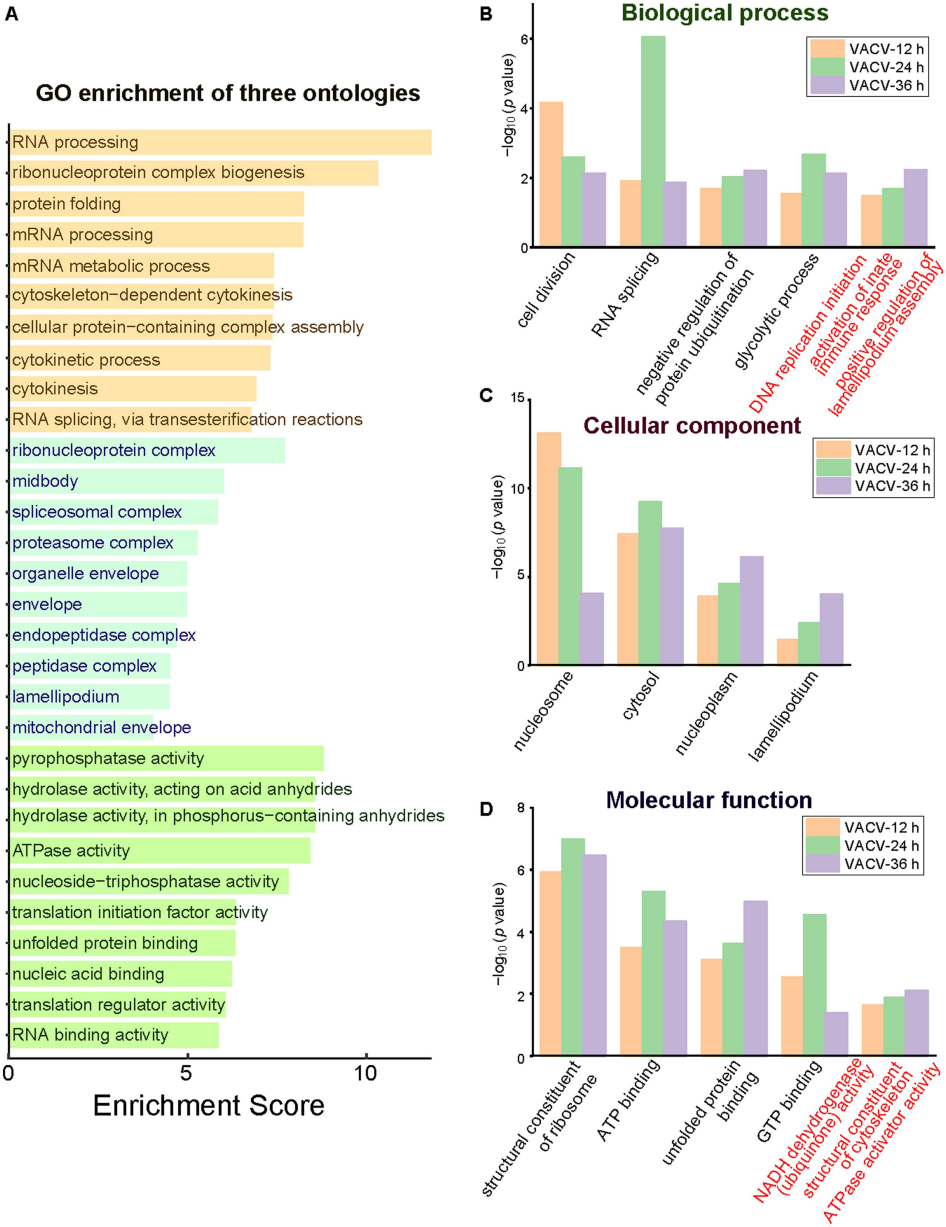
The GO enrichment of three ontologies at multiple time points of infection. **(A)** The holistic bar chart of the top 10 enriched terms in each ontology. The bar chart of GO enrichment in biological process **(B)**, cellular component **(C)**, and molecular function **(D)** at different time points of infection. Distinguished terms of each ontology are marked in red.

Functional and dysregulated molecules can be divided into categories of biological process, molecular function, and cellular components. GO terms offered a holistic insight into the enrichment of three ontologies to better understand the temporal differences during variable infection stages. The general layout of enriched dysregulated proteins listed the top 10 terms of each ontology (Figure 5A). A few critical terms have been enriched in biological processes, such as cell division, RNA splicing, negative regulation of protein ubiquitination, and glycolytic process (Figure 5B), indicating post translational modification regulation during VACV-induced cell migration. Exceptional biological terms at different time points of infection represented mutative cell manners during infection stages. DNA replication was initiated at the early stage to engage host components for completing viral life cycles (Weitzman and Fradet-Turcotte, 2018). The activation of innate immune responses was enriched particularly at the most significantly dysregulated time point of infection, which was consistent with poxvirus-induced immunopathological changes and potential biomarkers of disease progression (Li et al., 2023). Rapid cell motility depends on the formation of lamellipodia induced by the VACV F11L gene which facilitates migration (Morales et al., 2008). The achieved results suggested variable demands of host cell manipulation of biological processes during multiple stages of virus infection to accomplish viral life cycles.

During VACV-induced cell migration, terms of nucleosome, cytosol, nucleoplasm, and lamellipodium were enriched in the cellular component category (Figure 5C). In the molecular function category, the structural constituent of ribosome, ATP binding, unfolded protein binding, and GTP binding were common significant terms in multiple stages of VACV infection (Figure 5D). The energetic associated molecular functions indicated high energy demands during persistent VACV infection, especially in a series of energy-consuming processes of virus-host interactions (Kumar and Rangarajan, 2009; Mazzon et al., 2015; Sumbria et al., 2021). The results of GO and KEGG both revealed that VACV hijacked metabolic pathways for high energy consuming processes such as viral replication and the further regulation of actin cytoskeleton. Temporal proteomic profiling illustrated that VACV manipulated functional pathways over various stages of infection and accelerated cell motility. Based on the enrichment of GO and KEGG terms, the protein–protein interaction network provided a consistent and deep insight into the crucial functions of dysregulated proteins in VACV-induced cell migration.

### 3.4. Protein–protein interactions during VACV-induced cell migration

**Figure 6:**
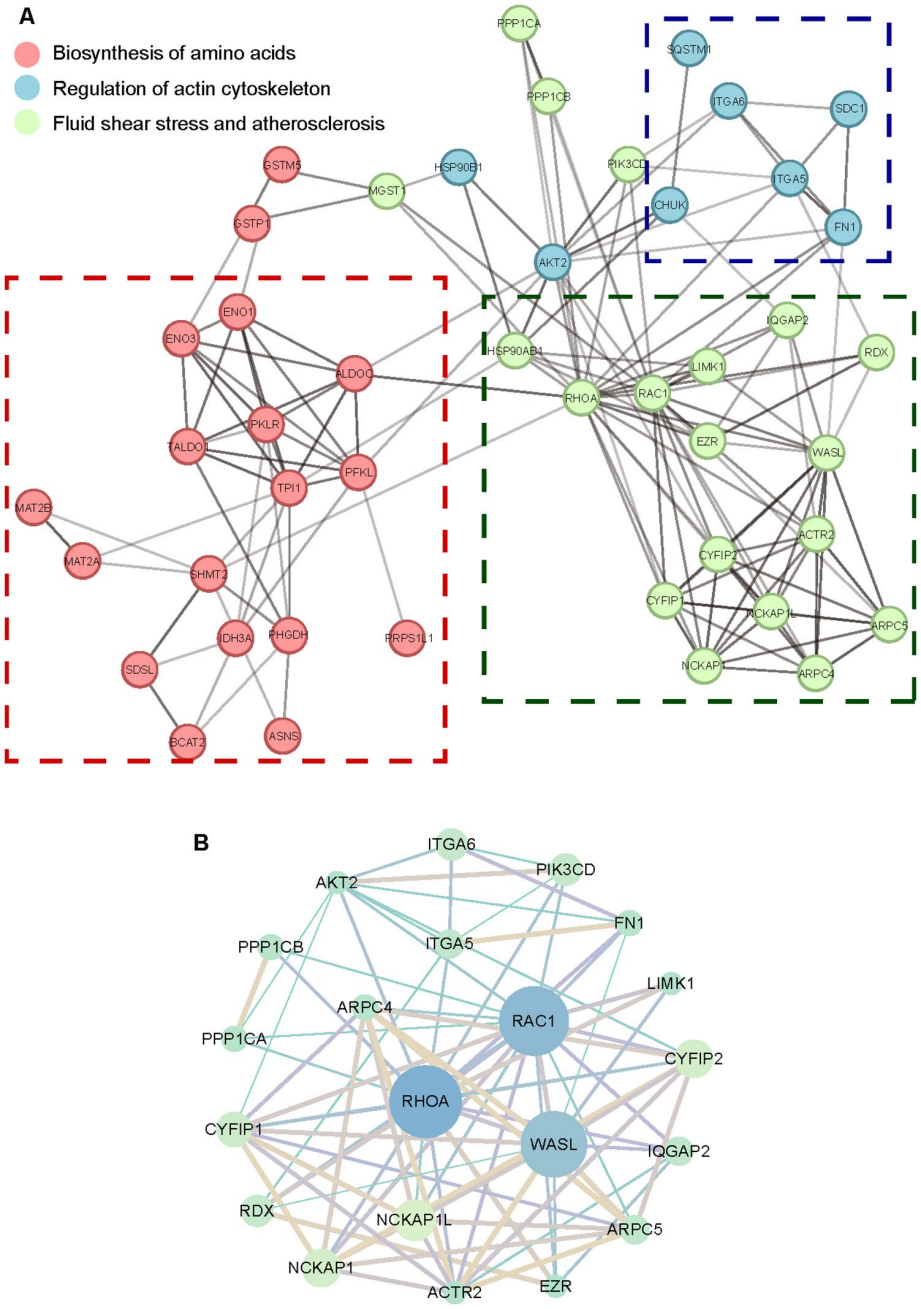
The protein–protein interaction network of dysregulated proteins. **(A)** The STRING network of dysregulated proteins distinguished by *k*-means. **(B)** The STRING network of the regulation of actin cytoskeleton. The circle size represents degrees of proteins in each cluster network. The shade of lines represents the combination score between various proteins.

To comprehensively understand the molecular interactions *via* functional proteins, the protein–protein interaction network by STRING was analyzed and specified. Dysregulated proteins were interacted through direct physical interactions and indirect function associations. The network is shown in Supplementary Figure S1. Part of the dysregulated proteins were classified into three clusters by k-means to group critical molecules of similar pathways and networks. Consistent with GO and KEGG enrichment consequences, the core proteins represented in each cluster were involved in the biosynthesis of amino acids, regulation of actin cytoskeleton, fluid shear stress, and atherosclerosis, respectively (Figure 6A). As reported in research on specific proteins of the small GTPase Rho family (Mercer et al., 2010; Handa et al., 2013), the significantly downregulated RhoA and Rac1 were centered in the interaction network of regulation of actin cytoskeleton (Figure 6B). This suggested the mechanism of Rho family-mediated skeletal morphological changes and corresponding net scattering dysregulated proteins.

In the biosynthesis of amino acids pathways, triose-phosphate isomerase 1 (TPI1) was in the center of the collaborated network (Supplementary Figure S2). As one of the critical glycolytic enzymes, TPI1 was observed to be upregulated in the presence of acute hepatitis C virus to stimulate increased flux through the glycolytic pathway (Diamond et al., 2010). With the exception of the regulation of actin cytoskeleton, RhoA was also involved and centered in the fluid shear stress and atherosclerosis (Supplementary Figure S3). Previous research has investigated small GTPase Rho-mediated signal transduction pathways activated by fluid shear stress (Lin et al., 2003), indicating multiple and irreplaceable functions of the Rho family in aberrant biological manners consisting of morphological and skeletal cell changes. Combining interaction network analysis with dysregulated protein scattering around each cluster offers a distinctive proteomic insight into investigating VACV-induced cell migration, targeted at discovering potential molecular biomarkers or for further biomedical and therapeutic treatments of viral diseases.

## 4. Conclusion

Our study utilized a temporal proteomic strategy to unravel unset dysregulated biological pathways during host-virus interactions. The identification and collaboration of critical molecules focused on functional metabolic pathways and highlighted distinctive functions of dysregulated proteins. Compared with conventional targeted analysis strategies, the temporal proteomic profiling provided an unbiased discovery of crucial proteins and their functional interaction networks. In summary, this work revealed a systematic and large-scale profiling of changing proteomes in VACV-infected host cells from a holistic perspective and complemented previous research on virus-induced cell migration. Also, the comprehensive understanding of protein dysregulations provides us with potential therapeutic targets and general biomedical applicability for viral treatments.

## Data availability statement

The datasets presented in this study can be found in online repositories. The names of the repository/repositories and accession number(s) can be found in the article/Supplementary material.

## Author contributions

JL and WL designed and accomplished all the experiments, also implemented data analysis, and wrote the original draft of this manuscript. X-ZC and YW assisted in the data analysis. TY assisted in virus experiments. BL, Y-JL, and LQ supervised, reviewed the manuscript, and acquired funding support. All authors contributed to the article and approved the submitted version.

## Funding

This study was supported by grants from the National Natural Science Foundation of China (Nos. 21934001, 22274026, and 31870978).

## Conflict of interest

The authors declare that the research was conducted in the absence of any commercial or financial relationships that could be construed as a potential conflict of interest.

## Publisher’s note

All claims expressed in this article are solely those of the authors and do not necessarily represent those of their affiliated organizations, or those of the publisher, the editors and the reviewers. Any product that may be evaluated in this article, or claim that may be made by its manufacturer, is not guaranteed or endorsed by the publisher.
