# Supplementary material for: Temporal proteomic profiling reveals functional pathways in vaccinia virus-induced cell migration
Source: Front Microbiol. 2023 Apr 25;14:1185960. doi: 10.3389/fmicb.2023.1185960 (PMC10249495; doi:10.3389/fmicb.2023.1185960)
Supplement: Supplementary file 2 [file Data_Sheet_2.docx]

Supplementary Material

Temporal proteomic profiling reveals functional pathways in vaccinia virus-induced cell migration

Jiayin Lu^1†^, Wei Liu^1†^, Xue-Zhu Chen^1^, Yiwen Wang^1^, Tianlei Ying^2^, Liang Qiao^1*^, Yan-Jun Liu^1*^, Baohong Liu^1*^

*** Correspondence:** Liang Qiao: [liang_qiao@fudan.edu.cn](mailto:liang_qiao@fudan.edu.cn);

Yan-Jun Liu: [Yanjun_Liu@fudan.edu.cn](mailto:Yanjun_Liu@fudan.edu.cn);

Baohong Liu: bhliu@fudan.edu.cn

# Supplementary figures

**
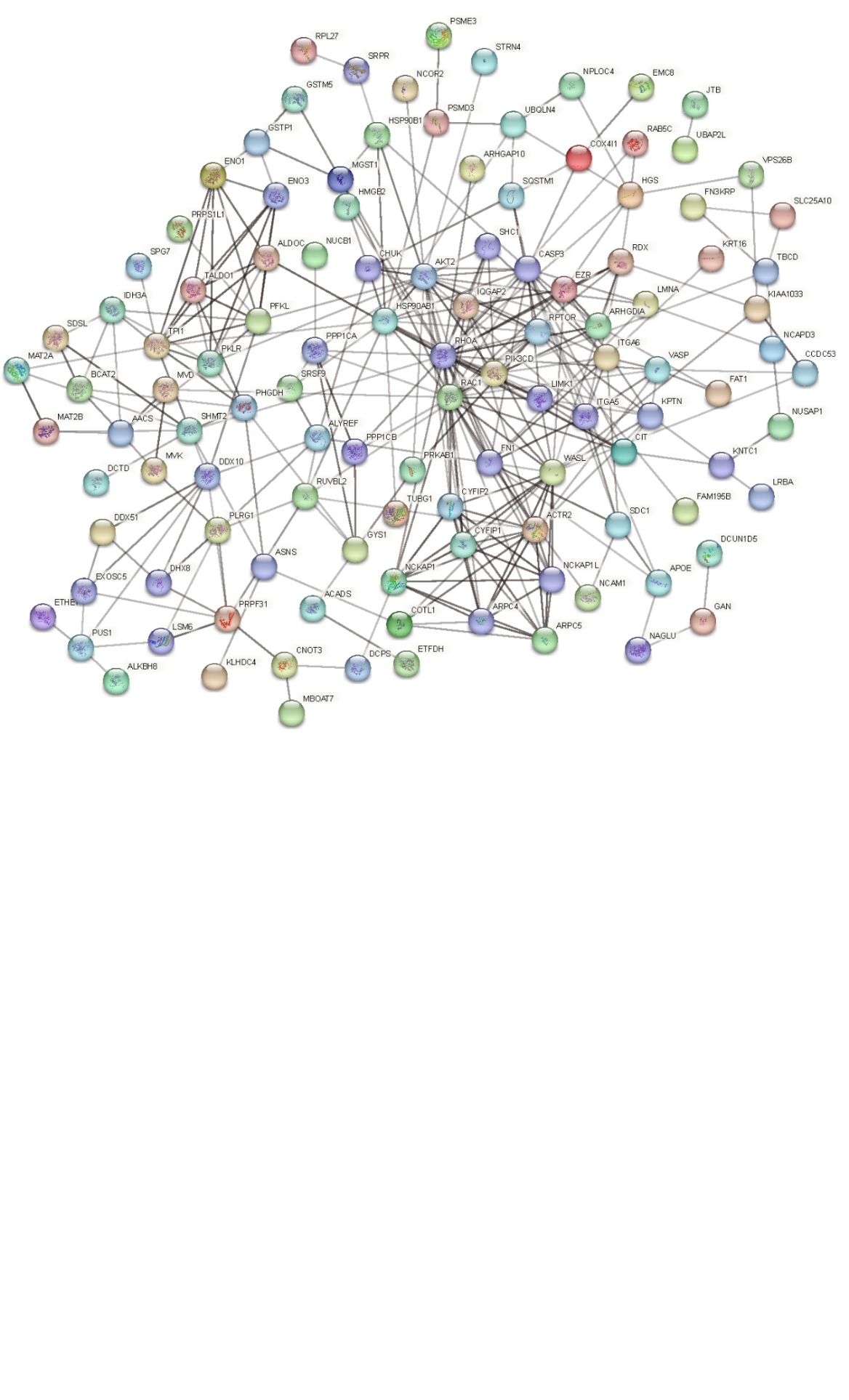
**

**Supplementary Figure S1.** The protein-protein interaction network of dysregulated proteins at VACV 24 h.p.i..

**
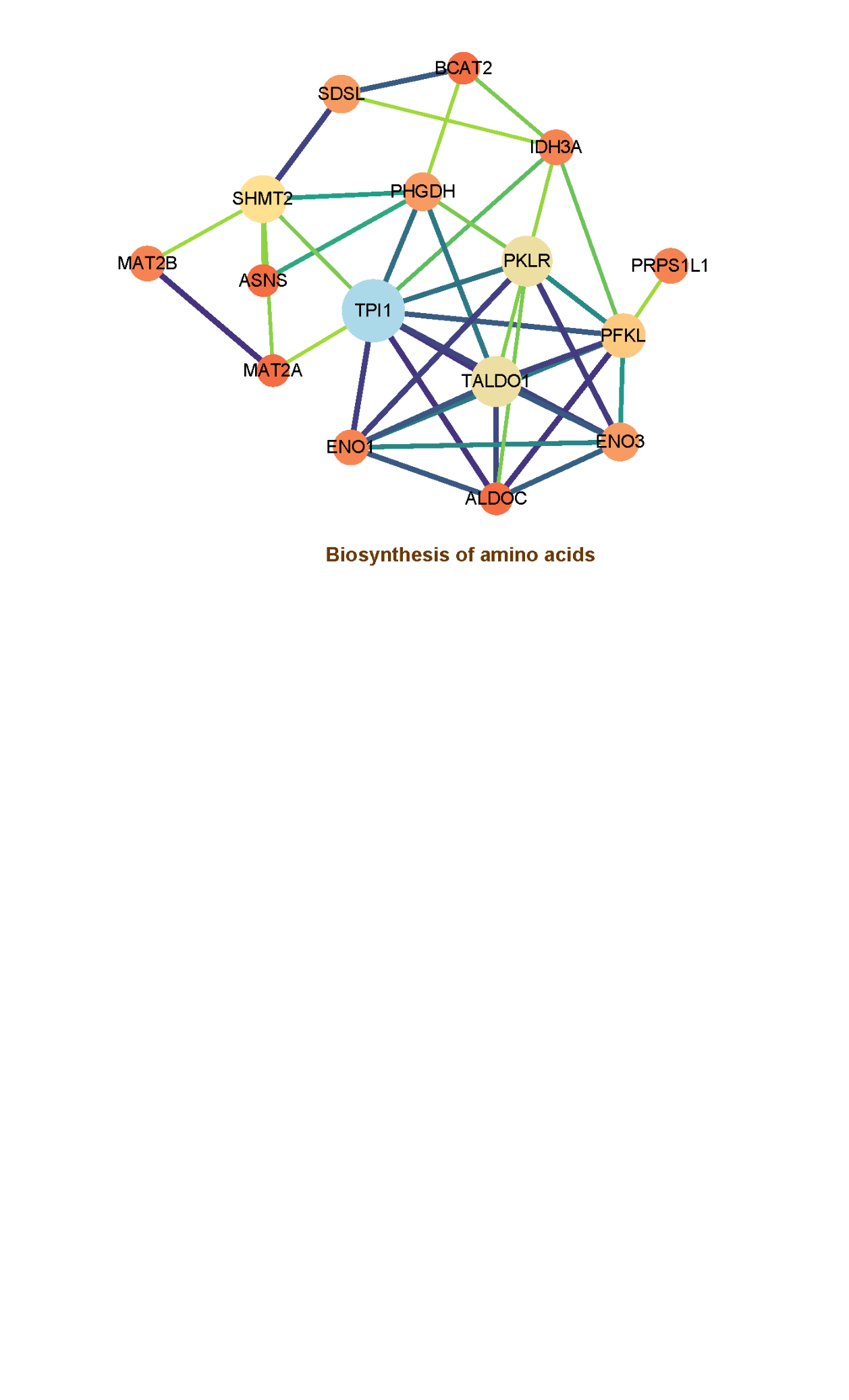
**

**Supplementary Figure S2.** The protein interaction network of biosynthesis of amino acids. Gradient color represents significant degrees of each molecule. The width of lines represents the combination score between proteins.


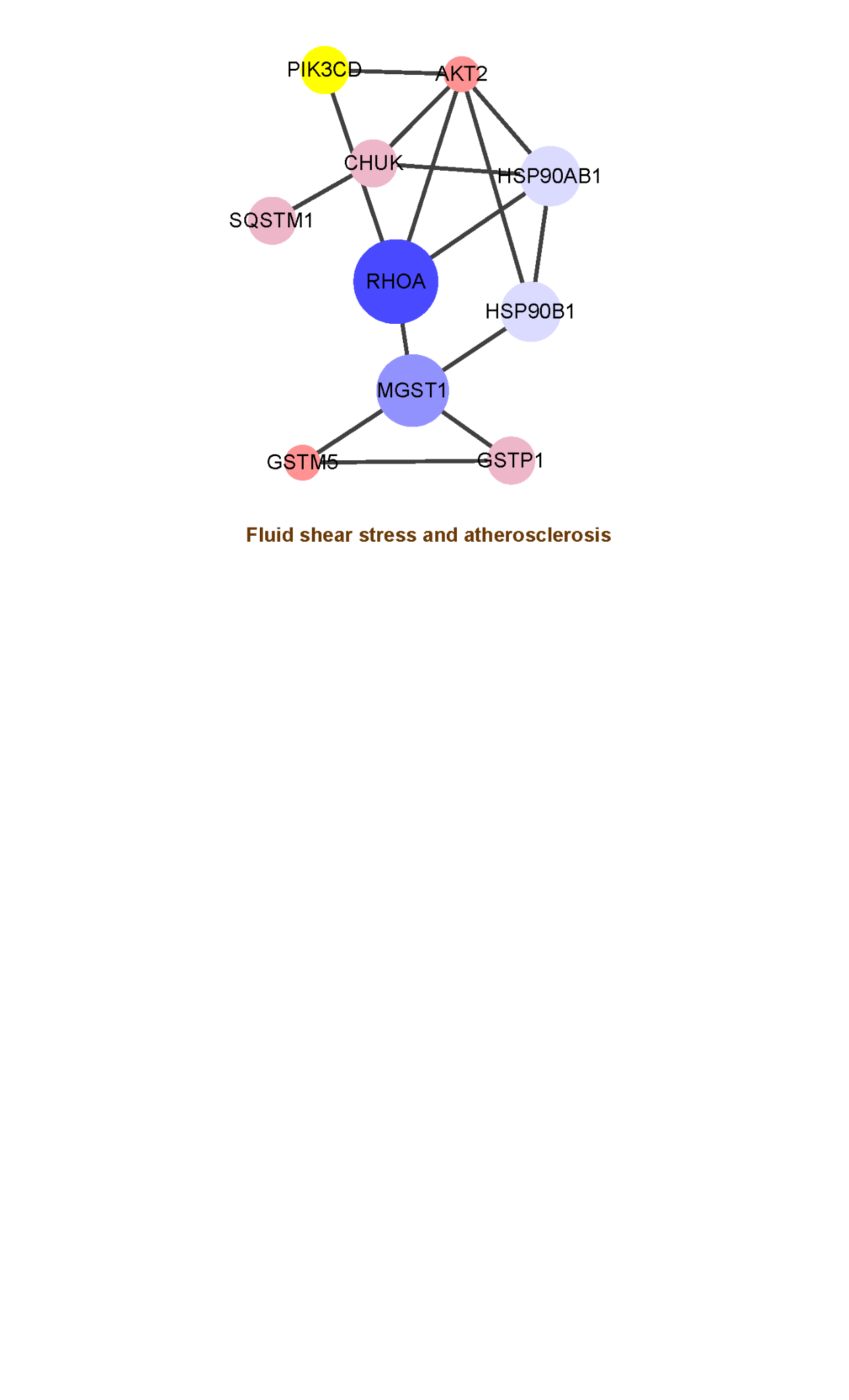


**Supplementary Figure S3.** The protein interaction network of fluid shear stress and atherosclerosis. Gradient color represents significant degrees of each molecule. The width of lines represents the combination score between proteins.

# Supplementary Datasets

**Dataset S1** (separate file). The identified proteins and raw intensities by temporal proteomics of non-infected and VACV-infected Vero cells at 12, 24, 36 h.p.i., respectively.

**Proteomics data**: The proteomics data have been deposited to the ProteomeXchange Consortium via the PRIDE (Perez-Riverol et al., 2022) partner repository with the dataset identifier PXD040845.

# References

Perez-Riverol, Y., Bai, J., Bandla, C., Garcia-Seisdedos, D., Hewapathirana, S., Kamatchinathan, S., et al. (2022). The PRIDE database resources in 2022: a hub for mass spectrometry-based proteomics evidences. *Nucleic Acids Res*. D1 (50), D543-D552. doi: 10.1093/nar/gkab1038
